# Long-Term Culture of Intestinal Cell Progenitors: An Overview of Their Development, Application, and Associated Technologies

**DOI:** 10.1007/s40139-016-0119-1

**Published:** 2016-10-12

**Authors:** Andrew J. Hollins, Lee Parry

**Affiliations:** 1 School of Biosciences, Cardiff University, Cardiff, CF10 3AX UK; 2 European Cancer Stem Cell Research Institute, School of Biosciences, Cardiff University, Cardiff, CF24 4HQ UK

**Keywords:** Intestinal, Organoid culture, Intestinal cell progenitors, Cancer stem cell, Disease models, Colorectal cancer

## Abstract

**Purpose of Review:**

Long-term culture of adult progenitor cells in 3D is a recently emerging technology that inhabits the space between 2D cell lines and organ slice culture.

**Recent Findings:**

Adaptations to defined media components in the wake of advances in ES and iPS cell culture has led to the identification of conditions that maintained intestinal cell progenitors in culture. These conditions retain cellular heterogeneity of the normal or tumour tissue, and the cultures have been shown to be genetically stable, such that substantial biobanks are being created from patient derived material. This coupled with advances in analytical tools has generated a field, characterized by the term “organoid culture”, that has huge potential for advancing drug discovery, regenerative medicine, and furthering the understanding of fundamental intestinal biology.

**Summary:**

In this review, we describe the approaches available for the long-term culture of intestinal cells from normal and diseased tissue, the current challenges, and how the technology is likely to develop further.

## Introduction

In 2007, after decades of research, the intestinal cell progenitors (ICPs) that are responsible for regenerating the surface of the normal intestine every 2–7 days were identified and shown to reside within crypt bases of the intestinal epithelium [1]. Up to this point, a variety of approaches had been used in an attempt to grow this tissue in vitro, including 2D immortalized cell lines often derived from either benign or malignant tumours, short-lived primary tissue isolates, as animal xenografts, and tissue pieces/slices. Each system not only had strengths but also shortcomings such as limited population doublings (primary isolates), ethical concerns (xenografts), or short-term viability (tissue slices). Following the identification of the ICPs, there was a period that saw major advances in the understanding of basic stem cell biology and refinements of progenitor cell culture. In 2007, it was demonstrated that 3D spheroids derived from CD133+ intestinal cancer stem cells (CSCs) could be maintained by utilizing culture medium refinements from neurosphere culture methods [2]. This formed part of a progression of developments, mainly pioneered by researchers then based within the laboratory of Hans Clevers, that in 2009 led to the identification of *Lgr5* as an ICP marker and publication of a 3D culture technique which allowed single murine intestinal stem cells to be grown into organoids that contained protruding crypt structures with all the cell lineages that comprise the small intestinal crypt in vivo [3, 4, 5•]. These cultures were grown in a mesenchyme-free environment comprised Matrigel (a reconstituted basement membrane gel [6]) in a medium with three organoid supporting supplements: epidermal growth factor (EGF); Noggin, which is a BMP signalling inhibitor that maintains an undifferentiated state; and R-Spondin, a modulator of the Wnt pathway and potent stimulator of adult stem cell proliferation [7]. The generation of mice harbouring an *Lgr5* driven GFP reporter [8] has enabled work that further characterized the crypt niche [9] along with identifying other ICP markers, notably *Bmi1* [10], and indeed proved crucial to the identification of R-Spondin as a key modulator of Wnt signalling. It was later observed that cultures of mouse colonic epithelium required the addition of Wnt3A to enable their indefinite expansion, suggesting that the organoid Wnt ligand production is insufficient to maintain colonic stem cells [11]. This work was then successfully translated into patient-derived ICP containing organoids utilizing similar media, although human intestinal and colonic organoids required both p38 and TGF-β inhibition (to suppress differentiation), with human colon culture additionally requiring Wnt3A, Prostaglandin E_2_ (that promoted organoid integrity through blocking anoikis and promoting proliferation), and Nicotinamide (a vitamin shown to inhibit differentiation) [11, 12].

This review discusses the progress made over the last 3 years in using organoid culture of tissue-derived ICPs. Related developments in which intestinal cultures are generated by the directed differentiation of embryonic or induced pluripotent stem cells are described and reviewed elsewhere [13–16]. Within this review, we will introduce the areas in which long-term tissue-derived ICP cultures are finding utility; (1) their application in studying disease processes (particularly CSC biology), (2) the prospective clinical applications of long-term ICP culture models, (3) the ongoing cell culture refinements and elaborations of ex vivo ICP models, and (4) an overview of the analytical technologies around the use of ICP organoids that will lead to the proliferation of ICP organoid platforms.

## Study of ICPs in Disease

ICP-generated 3D organoids retain in vivo cell-to-cell contacts, mass transport properties, mechanical properties, and metabolic profiles, whilst incorporating many cell types, modelling cell proliferation/differentiation, combined with long-term genomic stability [17•] and gene expression patterns. Thus, the organoids maintain their integrity, unlike classical 2D cell culture with its inherent loss of heterogeneity and the genomic rearrangements associated with the culture ‘crisis’/cellular senescence events that occur during cellular adaption. This maintenance of cell identity and genetic integrity within ICP containing organoid cultures makes them the current gold standard tool for interrogating basic and diseased intestinal biology ex vivo and the protocols for isolation of human intestinal progenitor cells from resected surgical samples and biopsies are now well established [18, 19]. Indeed the derivation of ICP organoid cultures from normal tissue and tumour material is carried out in such a way that cells are never grown directly upon culture plastic, as opposed to spheroid or tumoursphere culture models that are generated from established 2D cell lines. These organoid cultures have been particularly used in the study of colorectal cancer (CRC), and are being applied to translational settings such as regenerative medicine, diagnostic tests, and disease modelling [20–24].

### CRC Modelling Using Ex Vivo ICP Culture

Ex vivo ICP culture has been highly relevant in CRC, where the ISC has been identified as the cell of origin [25]. Previously, cancer research has relied heavily on the use of genetically modified mouse models (GEMMs) to explore the genes and pathways associated with the disease. However, these models predominantly develop tumours in the small intestine and not the large intestine, the reverse of the human situation. Indeed, for the time being ex vivo ICP-based culture systems do not yet fully recapitulate in vivo 3D architecture, nor the contributions of the stroma, endothelial cells, oxygen tension, blood supply, immune system, or innervations that are afforded by CRC GEMMs. Further, the herbivorous mouse small intestine microbiome bears little resemblance to the omnivorous human large intestine microbiome and does not model the significant role that the environment plays in CRC risk. Despite these current drawbacks, the potential major benefit of long-term ICP culture will be the reduction in the current reliance on GEMMs and a shift towards using human and mouse organoid cultures and systems that will increasingly reflect the in vivo CRC environment. Indeed, establishing reliable sources of this material is now crucial for researchers to understand normal and malignant ICPs. The following section will summarize the recent developments in using diseased ICP cells that have either come from patients, GEMMs, or that have been ‘engineered’/gene edited from one of the first two sources.

#### Patient-Derived ICPs

There are a number of paths to modelling CRC based upon the culture of ICPs, perhaps the most direct involving the collection of patient tumour material and the preparation of stable long-term cultures. This has led to the development of CRC organoid collections (or biobanks) which have the aim of representing the diversity of CRC disease, infection, and drug responses that exists within the patient population [17•, 26, 27]. An issue with such an approach is the need to develop a biobank that truly reflects the main disease subtypes. There are currently four clinical subtypes of CRC that have been recently assigned by the CRC Subtyping Consortium, they are microsatellite instability immune (CMS1; characterized by hyper-mutated, microsatellite unstable and strong immune activation), canonical (CMS2; epithelial, with marked Wnt and Myc signalling activation), metabolic (CMS3; epithelial and evident metabolic dysregulation), and mesenchymal (CMS4; prominent TGF-β activation, stromal invasion, and angiogenesis) [28]. The interpretation of drug response studies is notably more complex in the CMS1 subtype because whilst the genetic changes will classify the cultures the impact of immune cell components is missing from the assay readouts [17•]. Such collections require not only enough patients per subtype to power future analysis, but also culture conditions that address concerns regarding any selective bias favouring one CRC subtype over another. The key advantages of patient-derived ICP cultures are the capture of genetic combinations that are known drivers of human disease, the maintenance of disease associated epigenetic history, and the ability to recapitulate the histology of primary disease.

#### GEMM-Derived ICPs

The second major source of malignant ICP cultures is from genetically characterized GEMMs. Despite the drawbacks previously mentioned, they are still invaluable tools for research and can be combined with organoid culture to great effect. This was epitomized by the work from the group of Doug Winton, who used ICPs from GEMMs to generate organoids that could contain a singly mutated ICP to understand the altered crypt dynamics and clonal advantage induced by the most common genetic alterations pervading CRC biology (Apc loss, Kras activation, and P53 mutation) [29]. Indeed over the past 3 years, GEMMs and organoids have been used to investigate the myriad of ISC- and CRC-associated genes and their relevant pathways to identify which are driving the tumour and which are passengers, e.g. *Troy* [30], *Brg1* [31], *Cdx2* [32], *Kcnq* [33], *Prox1* [34], *Fzd7* [35], *Yap* [36]. As well as the continuing exploration of intestinal biology, the GEMM-derived organoids are also being deployed as platforms to screen how normal gut and primary tumours with defined mutations respond to potential cancer therapeutics and determine the mechanisms of action. For example, Lorenzi et al. have used this approach to demonstrate that the resistance of *FBXW7*-mutated CRC cells to certain types of chemotherapy (e.g. Fluorouracil (5-FU)) is due to an inhibition of terminal differentiation indicating the that they could be overcome by using differentiating therapies [37]. ICP culture has been used to investigate the p300-CREB-MYB protein interactions and its role in Oxaliplatin resistance [38]. It has been demonstrated that the changes in gene expression pattern in a malignant intestinal stem cell are also closely tied to radiation resistance; Ladang et al. identified that the expression of *Elp3* plays a key role in the radio-resistance of the Lgr5^+^Dclk1^+^ malignant ISC due to its promotion of *Sox9* translation [39]. There are of course limitations to GEMM work that are driving the development of the human CRC models described above, such as the costs associated with their creation, their maintenance, and their inability to accurately reflect the biology of the human large intestine, particularly as the majority of GEMM studies on intestinal tumourigenesis have performed in the mouse small intestine. A key advantage going forwards will be the ability to reduce the cost of GEMM studies through combining defined genetic backgrounds with the ability to establish them as long-term organoid cultures and in doing so obtain much more data per animal.

#### Genome Editing of ICPs

A third approach capable of utilizing both of the above CRC culture derivations is through the exploitation of current DNA manipulation technologies to manipulate normal mouse and human ICPs into cancer ICPs that reflect human CRC. Using shRNA to target APC, P53, and PTEN, Onuma et al. have used lentiviral vectors in normal ICPs that has enabled them to generate recapitulated intestinal tumour organoids without generating gene-modified mice [40]. Similarly, Wang et al. [41] using adenovirus vectors and Ju et al. using food derived exosome delivery of nanoparticles [42] have provided proof of principle studies to demonstrate these techniques as effective gene delivery vehicles for genetic manipulation in 3D organoid cultures. However, future work is likely to use genome editing technology to a greater extent. The groups of Toshiro Sato and Hans Clevers have pioneered the use of CRISPR-Cas9 in ICP culture to demonstrate that common CRC mutations (often termed as ‘driver mutations’) confer niche-independent stem cell maintenance but not to metastatic progression, with data indicating that additional molecular lesions are also necessary for invasive tumour behaviour [43•]. A key feature of these techniques is the ability to modify genes in a stepwise fashion enabling the immediate analysis of the effect each gene has on an ICP [40, 44]. ICP culture has demonstrated that oncogenic alterations activating the MAPK and Wnt/β-catenin pathways must be consecutively and coordinately selected to assure stem cell maintenance during colon cancer initiation and progression [45]. Germann et al. [46] used organoids generated from *Apc* ^*min*/+^ mice noting an aberrant cyst-like sphere morphology induced by a constitutively activated Wnt pathway that was responsible for increasing both self-renewal and growth while reducing differentiation. This observation combined with deletion studies elegantly described the engagement of the Wnt, Notch, and Myb transcriptional pathways in intestinal tumourigenesis and further highlighted the Wnt pathway as a therapeutic target in CRC. It seems likely that these last techniques will slowly lead us away from our current reliance on GEMMs.

## Clinical Applications of Long-Term ICP Culture Models

This section summarizes in turn, personalized medicine, drug discovery, and regenerative medicine, the three main areas of clinical application for long-term ICP cultures.

### Personalised Medicine

**Fig. 1:**
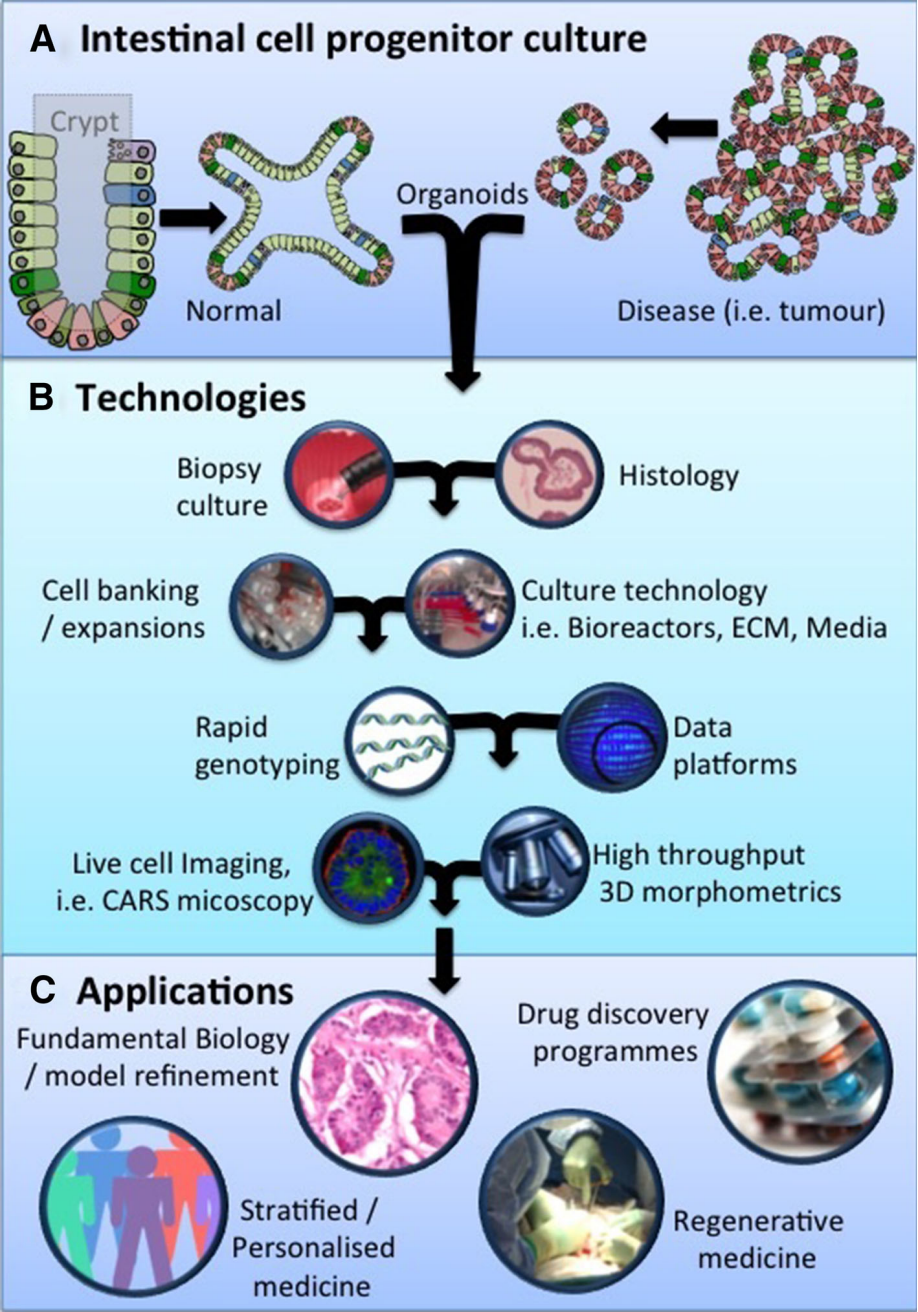
Schema showing the scope of tissue-derived ICP culture. **a** Intestinal cell progenitor culture, the origin of different organoids (normal and disease). **b** A collection of techniques and technologies that need to be in place in order to fully exploit the potential of ICP long-term cultures, for example the need for rapid genotyping to ensure the integrity of cultures from passage to passage. **c** The main applications and areas of ongoing research for potential deployment long-term ICP cultures in biomedical and clinical settings

One of the greatest potential benefits of ICP cultures is to deliver a personalized medicine paradigm, where much like a biomedical service such as microbiology or virobiology a small sample is supplied and then cultured for rapid analysis. Where cultures act as ‘avatars’ of patients in the dish, an emerging application recently summarized by [47] allows clinical feedback of sample response to available therapeutics (Fig. 1). Ideally, these samples could also seed the biobanks described in the previous section, increasing the potential to break the existing 2D cell line paradigm that is prevalent in commercial drug/toxicity testing [48].

### Drug Discovery

Diverse collections of patient samples reflecting population disease profiles may power future drug discovery programmes, wherein organoids representative of specific disease subtypes would be tested against panels of compounds in drug titration assays in order to determine their potential efficacy. Key to shifting the current 2D culture led paradigm will be the generation of defined batches of organoids through scalable processes for commercial drug discovery programmes. Already the use of ICP culture is currently expanding knowledge of the role of individual genes in response to injury [49], and chemical-induced injury, e.g. *ID1* [50].

### Regenerative Medicine

Increasing our understanding of the influence of diet on the ISC using ex vivo culture is identifying the mechanisms for understanding the cause of disease resistance and simultaneously opening up potential avenues to be exploited for regenerative medicine. Potentially, the relationship between a high fat diet (which has been shown to increase the self-renewal potential of intestinal organoids), and susceptibility to CRC could be exploited to aid regeneration following intestinal injury [51]. Further, it has recently been demonstrated that transplantation of ICP organoids can potentially be used to increase the absorptive area in patients with short bowel syndrome [52] or alternatively tissue reconstruction, i.e. bowel reconstruction after disease, could be achieved using de-cellularized scaffolds for growing functional epithelium. The tools of organoid culture have also been explored in single-gene hereditary defects affecting the intestine, notably in studies of the cystic fibrosis transmembrane conductor receptor where organoids derived from the ISCs of cystic fibrosis patients have facilitated functional studies, drug development, personalized medicine, and gene repair approaches to treating the disease [53–55]. Gene manipulation in vivo and ex vivo has led to the conversion of ICPs into insulin producing “neo β-cell islets”, providing a potentially abundant and accessible source of functional insulin producing cells [56].

However, for organoids to achieve their clinical potential, biobanks of intestinal disease (i.e. CRC) would ideally (1) contain the tumour, blood (germline DNA), and early passage organoid set for DNA/expression analysis, which would allow the checking for a faithful recapitulation of the tumour by the organoid culture, alongside the identification of mutations from polymorphisms, (2) the ability to supply/maintain organoids in sufficient quantities and under standardized conditions to facilitate drug titration assays, which will require the development of bioreactors capable of standardizing growth and assay material, and (3) a sample collection reflecting a broad genetic diversity enabling toxicity studies where the range of likely toxic responses can be monitored.

## Advances in Ex Vivo ICP Culture

Increasingly, the literature demonstrates a proliferation of the use of, and the number of, applications of ICP models (Table 1). Indeed, there have been some attempts to define and standardize conditions through publications of detailed protocols [57], and commercial production of specific media. Further streamlining of key components of the culture platform will be required to generate fully defined and reproducible growth conditions, a key example is the 3D support/extracellular matrix. The most commonly used support matrix, Matrigel, is a biological derived product that has a protein matrix composition of laminin, entactin, collagen, and heparan sulphate proteoglycans with batch variations compounded by varying concentrations of growth factors, such as bFGF, EGF, IGF-1, PDGF, NGF, and TGF-β [58]. Despite the work of some groups to date a defined hydrogel suitable for long-term ICP culture has not been reported, matrix biologists from the field of directed stem cell differentiation are now engaging with organoid culture [59].

**Table 1:** Overview of intestinal cell progenitor culture model applications ranging across many areas of intestinal biology

Subjects	Area of investigation	Tools/technology	Culture	Species (tissue)	Publication/s
Tumour biology	Modelling colorectal cancer progression	CRISPR-Cas9 introduction of APC, KRAS, SMAD4, TP53 and PIK3CA	Wnt, R-spondin, epidermal growth factor (EGF), noggin, transforming growth factor (TGF)-β inhibitors	Human	Matano et al. [43•]
	Oncogenic BRAF induced loss of intestinal stem cells is antagonized by β-catenin activity	GEMM BRAF^V637E^ knock-in mice	Varied	Mouse	Riemer et al. [45]
	Interleukin-22 promotes intestinal-stem-cell-mediated epithelial regeneration	GEMM LGR5-GFP reporter mice Lgr5-LacZ reporter mice	Co-culture with innate lymphoid cells Defined EGF; Noggin, R-spondin organoid media	Human and mouse	Lindemans et al. [76]
	Organoid metabolism, particularly aspects of the Warburg shift in tumorigenesis	Lgr5-EGFP-IRES-creERT2 mice Measuring energy metabolism	Defined BMP antagonist LDN-193189, EGF, R-spondin, Wnt conditioned medium	Mouse	Fan et al. [74]
Regenerative medicine	Short bowel syndrome Small intestine reconstruction	Culture onto a polyglycolic acid scaffold	Organoids cultured on scaffolds DMEM, 10 % serum	Human and mouse cultures into NOD/SCID mice	Grant et al. [52]
	Colonic mucosal injury	EGFP labelled donor mice	Defined EGF, Noggin, R-spondin organoid media	Mouse into mouse model	Fukuda et al. [49]
Intestinal stem cell biology	A high-throughput platform for stem cell niche co-cultures and downstream gene expression analysis	Lgr5^EGFP-CreERT2^ mice Sox9^EGFP^:CAG^DsRed^ Single cell microfluidics platform to interrogate stemness, transcriptional characterization of niche lineages	Single cell culture AdvDMEM, N2, B27, Nac, 5 % FBS	Mouse	Gracz et al. [67]
	Studying cdx2 and its role in intestinal progenitor cell lineage Transformation of intestinal stem cells into gastric stem cells on loss of Cdx2	Lgr5-EGFP-Ires-CreERT2 and Cdx2+/− mice	Intestinal organoids EGF, Noggin, R-spondin Stomach organoids EGF, Noggin, R-spondin, Wnt, Fgf, Gastrin	Mouse	Simmini et al. [32]
	Metabolism	Mapping early fate determination in Lgr5+ crypt stem cells using a novel Ki67-RFP allele	LGR5 cell cycling and Wnt signalling, ki67 labelled mouse model	Mouse	Basak et al. [9]
Infection models	Impact of bacteria on intestinal epithelial cell biology, i.e. infection and irritable bowel disease	*Salmonella* (co-culture)	EGF, Noggin, R-spondin based with study specific additions	Mouse Human	Zhang et al. [50] Rouch et al. [77]
		*Helicobacter pylori* Organoid microinjection	EGF, Noggin, R-spondin, Wnt, Fgf, Gastrin	Human (normal and tumour)	Bartfeld et al. [79]
		*Escherichia coli* (various pathogenic strains)	Wnt, R-spondin, Noggin	Human	VanDussen et al. [26]
Tissue engineering/co-culture models	Gastric epithelium stem cell maintenance	Co-culture Collagen gels	Myofibroblast cell line Collagen gel in an air–liquid interface environment, Ham’s F12, with 20 % FBS	Mouse (gastric)	Katano et al. [63]
	Intestinal crypt fission	Atomic force microscopy Lgr5-EGFP-ires-CreERT2 mice	EGF, Noggin, R-Spondin	Mouse (glandular stomach culture)	Pin et al. [62]

Aside from standardization of existing models, ongoing elaborations of ICP niche culture are being pursued in efforts to draw models still closer to recapitulating the organ in a dish (Table 1). There are a growing number of co-culturing methods to elaborate organoids with other cell types from the intestinal niche: (1) nerve and fibroblast cells replacing the need for exogenous Wnt signalling [60], myofibroblasts [61–63], gut nerve cells [64], (2) microorganisms of the gut microbiome modulating nutrient availability [65, 66], and (3) the use of microfluidics to explore niche dynamics [67, 68] (Table 1). Many of the above uses of these cultures are also discussed by Fatehullah et al. [69]. Again, the key is to refine culture techniques that accurately replicate the in vivo environment whilst addressing the challenge of establishing reproducible systems suitable for their application in drug discovery programmes.

## Analysis of ICP Organoids

The use of ICP culture and technology to alter gene behaviour can elicit a myriad of cellular biology; thus, there is a requirement for techniques that can be used to analyse and quantify relevant parameters within complex 3D cellular environments [19]. This section sets out the cell culture analysis tools that can be used with 3D organoid cultures to quantify the complexity of organoid systems that display a wider range of biology compared with ‘classical’ 2D culture models. In some cases, tools have been modified to better work in 3D culture conditions.

### 3D Organoid Visualization

A change from 2D to 3D biology represents a sizable image analysis challenge. Standard light microscopy image analysis (often achieved using whole well or plate scanning imaging apparatus) is limited to 2D images, offering relatively few extra parameters (i.e. organoid diameter, organoid area, and number of organoids), although they do facilitate non-destructive serial measurements. In order to exploit the readouts of commonly used immunohistochemistry and immunofluorescence methods (for example live/dead cell detection, cell polarity, cell lineages, and organoid architecture), microscopic imaging platforms, such as wide field and confocal high content microscopes, are being used to analyse organoid cultures in 3D. Software analysis pipelines that are capable of analysing hundreds of 3D morphometric parameters and readouts can be used to identify subtle biological effects of media conditions and drug treatments [70]. These workflows are being designed to be compatible with the medium- to high-throughput drug screening demands of the pharmaceutical industry, and have led the development of morphometric analysis tools able to handle and interpret the volume of image stacks generated. The power and continued evolution of these screening platforms will be needed if 3D ICP-containing tumour models are to replace 2D cell lines in the preclinical drug discovery process. Image analysis of organoids may also be aided by the development of label-free imaging approaches such as coherence anti-Stokes–Raman spectroscopy (CARS) since cell type identification within complex 3D cellular environments currently requires organoid fixation and permeabilization [71, 72]. With the enhanced microscopy platforms, such as CARS, it will very likely be the timely analysis and interpretation of massive volumes of data created that represent the rate-limiting step to their application in drug screening and discovery programmes.

### Single Cell Analysis

The ability to isolate and examine single cells within an organoid is an important feature of ICP culture. As the next-generation single-cell gene profiling becomes increasingly available in research labs, further progress will be made in understanding intestinal biology and disease. Existing next-generation sequencing technology has already been adapted and exploited to identify new intestinal cell types within organoids based on single cell messenger RNA sequencing [73]. This will allow lineage tracing experiments and a greater understanding of the dynamics of normal and diseased cells within the crypts. Although the readouts are still relatively limited, standard biochemical assays (live/dead ATP assays) have also been adapted for use in 3D culture, and other bioassay techniques are also coming on line. Techniques such as those reported by Fan et al. [74] wherein they adapted existing tools to create a platform capable of tracking dynamic energy metabolism in organoids and demonstrated a Warburg-like metabolic profile associated with colon tumourigenesis.

For the current rate of progress in the use of organoids to be maintained, it will be important that the development of technology, for obtaining and interpreting the vast quantities of information that can be gained from long-term ICP cultures, does not lag far behind.

## Bringing Ex Vivo Closer to In Vivo: The Next Challenge

The complexity of the intestinal niche necessitates further elaboration of culture model systems that will likely include gut microbiota and diet research. Current models do not include stroma, elements of the immune system, a disease-specific ECM, or gut bacteria (the latter applicable to drug development for infection models). The ability of ICPs to faithfully maintain physiological relevance over time is vitally important for their use as research tools. However, replicating the environment in the human large intestine is crucial to our understanding how CRC develops. Culture conditions that manipulate the signalling pathways essential for ISC function are continually being identified and refined to better replicate and understand the ISC niche [75, 76]. Recent research has taken a reductionist approach to begin understanding the enormous complexity of this system. Reports investigating the role of single components of the diet [51], microbiome [26, 77–81], metabolome [66], immune system [81], and stroma [63, 82] on ICP using ex vivo culture are starting to emerge. These have demonstrated an increased understanding of how the environment elicits cellular and epigenetic alterations [83, 84] that are relevant to human health and intestinal diseases. Beyaz et al., using an ex vivo model, recapitulated ex vivo the environment associated with a high fat diet and established a *PPARδ*-dependent link to an increase in stemness within the intestinal niche that predisposes to CRC. Although these reductionist approaches are yielding greater insight into ICP, ultimately there will need to be greater efforts made to bring ex vivo culture techniques closer to the in vivo environment. The challenge for the future is to develop the tools for ICP culture to the point where human intestine can be recapitulated in the laboratory, such as elaborate co-culturing systems involving microfluidic linked culture vessels mimicking multi-compartment and even multi-tissue interactions. This would enable the exploration of the interactions between the full range of factors (diet, microbiome, metabolome, stroma, and immune system) that impact on the ISC and the roles they play in promoting, preventing, initiating, and driving intestinal diseases.
